# Plant-expressed Zika virus envelope protein elicited protective immunity against the Zika virus in immunocompetent mice

**DOI:** 10.1038/s41598-023-47428-7

**Published:** 2023-12-27

**Authors:** Minna Shin, Hyangju Kang, Kyeong ryeol Shin, Rangyeon Lee, Kiju Kim, Kyungmin Min, Kyou-Nam Cho, Eun-Ju Sohn, Kwang Sung Kim, Seok-Hyun Kim, Yang Je Cho, Jeongho Park, Tae-Wook Hahn

**Affiliations:** 1 INNOVAC, Chuncheon, 24341 Republic of Korea; 2 BioApplications Inc., Pohang Techno Park Complex, 394 Jigok-Ro Nam-Gu, Pohang, Korea; 3 College of Veterinary Medicine & Institute of Veterinary Science, Kangwon National University, Chuncheon, 24341 Republic of Korea; 4 EYEGENE Inc., B-1211, 401 Yangcheon-Ro, Gangseo-Gu, Seoul, 07528 Republic of Korea

**Keywords:** Diseases, Infectious diseases, Viral infection

## Abstract

Zika virus infection causes multiple clinical issues, including Guillain–Barré syndrome and neonatal malformation. Vaccination is considered as the only strategy for the prevention of ZIKV-induced clinical issues. This study developed a plant-based recombinant vaccine that transiently expressed the ZIKV envelope protein (ZikaEnv:aghFc) in *Nicotiana benthamiana* and evaluated the protective immunity afforded by it in immunocompetent mice. ZikaEnv:aghFc induced both humoral and cellular immunity at a low dose (1–5 μg). This immune-inducing potential was enhanced further when adjuvanted CIA09A. In addition, antigen-specific antibodies and neutralizing antibodies were vertically transferred from immunized females to their progeny and afforded both protective immunity to ZIKV and cross-protection to Dengue virus infection. These results suggest that our plant-based ZIKV vaccine provides a safe and efficient protective strategy with a competitive edge.

## Introduction

Zika virus(ZIKV) infection is a vector-borne disease that is caused by a virus classified as a flavivirus in the *Flaviviridae* family, which also includes the dengue virus (DENV), West Nile virus (WNV), and yellow fever virus (YFV)^1,2^. ZIKV has remained a risk factor for public health for the last 30 years, with more than 1.5 million infections being reported in Brazil in 2015^3,4^. Patients with ZIKV infection may be asymptomatic or develop mild self-limiting disease; however, they may also exhibit severe neural disorders, such as Guillain–Barré syndrome^5,6^. Of note, ZIKV infection during pregnancy is closely associated with microcephaly and multiple neural signs in neonates^7^. However, clinical trials and preventive strategies have not been authorized for ZIKV^8^; thus, efficient and safe vaccine strategies against ZIKV infection are necessary.

The ZIKV genome encodes three structural proteins (the capsid (C), pre-membrane (prM), and envelope (E) proteins) and seven nonstructural proteins (NS1, NS2A, NS2B, NS3, NS4A, NS4B, and NS5)^9^. Each structural protein exerts its own function: the C protein binds to viral RNA, to constitute the nucleocapsid; the prM protein forms a complex with the E protein, which facilitates protein folding and prohibits premature fusion to host membranes; and the E protein plays critical roles in invasion and expansion in the host, such as viral assembly, attachment, entry, and fusion^10,11^. Numerous studies have utilized the E protein as a major target for vaccine development, because this molecule presents epitopes and induces neutralizing antibodies^12–16^.

Various types of ZIKV vaccines, such as DNA, subunit, inactivated, virus-vector-based, live-attenuated, virus-like particle (VLP), and mRNA vaccines, have been under development. The subunit vaccine is thought to be a promising protective strategy because it is a safe, stable, reliable, and cost-effective clinical tool^1,8^. Plants are an active bioreactor for protein production because they produce a wide range of proteins. Moreover, the production costs, capital investments, and infrastructure costs associated with plant-based protein production are low. In addition, plant-produced proteins are less likely to be contaminated by animal pathogens^17–19^. Therefore, efforts have been made to produce vaccines using plant expression systems. For example, vaccines for COVID-19, classical swine fever virus, and other pathogens were developed using plant-based systems ^20–25^.

Subunit vaccines are reliable, effective, and safe (even during pregnancy) and have fewer side effects^26,27^. However, a recombinant ZIKV subunit vaccine requires immunization with adjuvants because of its low immune-stimulating activity^8,28^. Aluminum salt (Alum) is widely used as an adjuvant because it affords reliability, antigen stabilization, and a durable antigen titer. Moreover, it stimulates the Th2 and humoral responses, but not cellular immunity, such as the Th1 response and cytotoxic T-cell induction^29–32^. In turn, monophosphoryl lipid A (MPL) is a Toll-like receptor 4 (TLR4) agonist that is derived from the *Salmonella minnesota* R595 strain and is a chemically detoxified derivative of the parent lipopolysaccharide (LPS)^33^. This adjuvant activates TLR4 and innate immunity, generates proinflammatory cytokines, and triggers the Th1 response^32,34,35^. Furthermore, CIA06 is prepared in combination with the TLR4 agonist, detoxified LOS (dLOS), and Alum and is active in various viral and bacterial vaccines^36–40^. For example, a phase I clinical trial of a CIA06-adjuvanted human papillomavirus (HPV) virus-like particle (VLP) vaccine is currently under way^41^. CIA09A consists of cationic LMP, a TLR4-agonist deacylated low-fat sugar, and cholagogue saponin fraction QS-21. Moreover, a CIA09A-adjuvanted *Varicella zoster* virus (VZV) glycoprotein E (gE) vaccine stimulated both humoral and cellular immunity against VZV^42^. The major goal of vaccine development is to provide a potent and prolonged immunity that is supported by fine-tuned adjuvant use.

Here, we used a plant expression system that targeted the ZIKV E protein to develop ZIKV recombinant subunits (ZikaEnv:hFc and ZikaEnv:aghFc). Multiple adjuvants, such as Alum, MPL, CIA06, and CIA09A, were evaluated together with these vaccine candidates. Subsequently, we determined the optimal dose and examined the levels of humoral and cellular immunity afforded by the vaccine candidates. As a result, we observed that pups from ZikaEnv:aghFc-immunized females exhibited protection against two ZIKV strains, MR766 and PRVABC59, and cross-protection against a Dengue viral strain. Therefore, this study provided a potent and stable ZIKV vaccine candidate.

## Results

### ZikaEnv:hFc and ZikaEnv:aghFc expression in N. benthamiana and their purification

**Figure 1:**
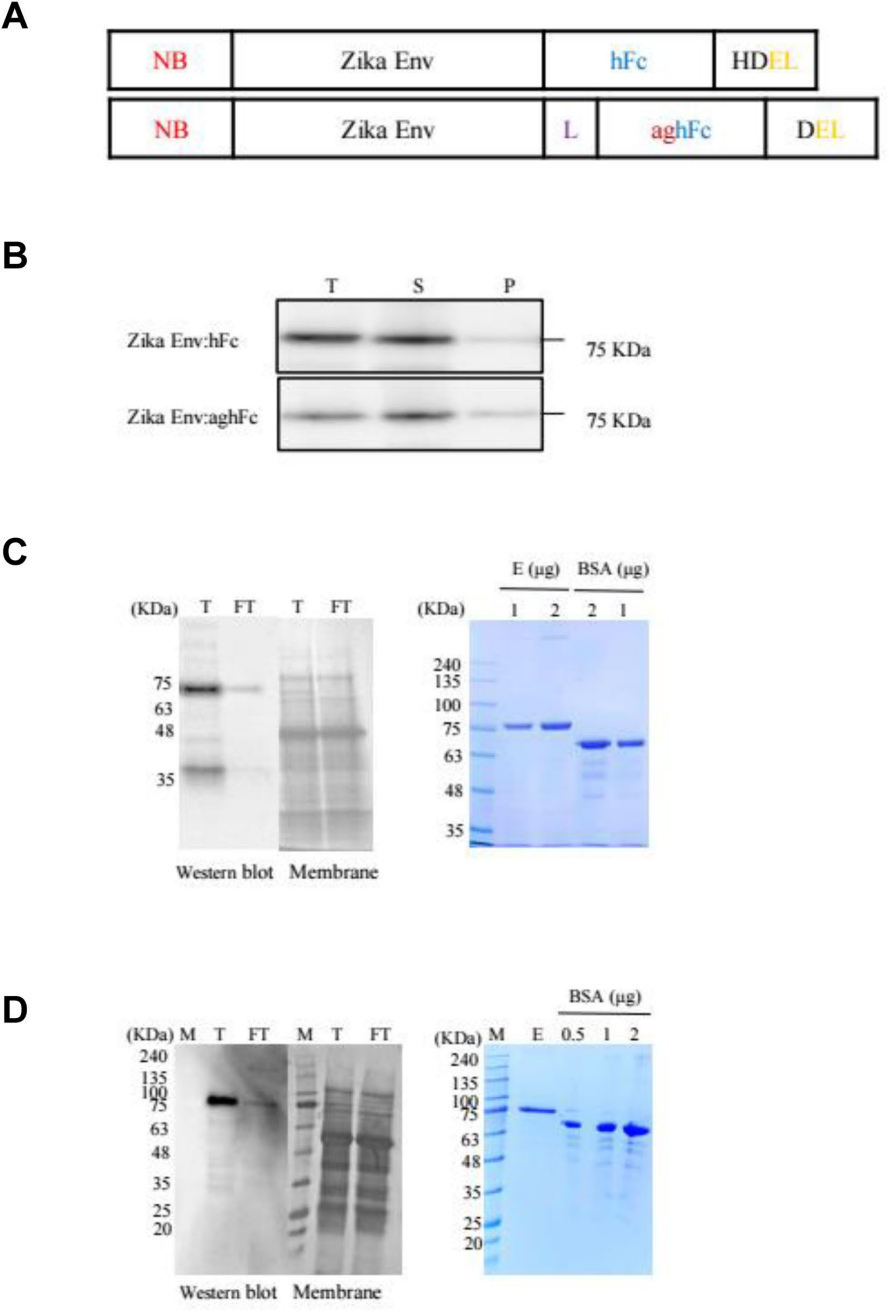
Expression and purification of Zika Env:hFc and Zika Env:aghFc. (**A**) Schematic representation of Zika Env:hFc and Zika Env:aghFc. NB, new chaperone-binding protein (signal peptide); hFc, constant fragment of human immunoglobulin G; HDEL, histidine–aspartate–glutamate–leucine peptide (endoplasmic-reticulum-retention signal); L, peptide linker (GGGGSGGGGS); aghFc, aglycosylated hFc; DEL, aspartate–glutamate–leucine peptide (endoplasmic-reticulum-retention signal with the last lysine from aghFc). (**B**) Expression and solubility of recombinant Zika Env:hFc and Zika Env:aghFc in *Nicotiana benthamiana*. Each fraction was subjected to Western blot analysis using an HRP-conjugated anti-human IgG antibody. T, plant total extract; S, soluble fraction; P, pellet fraction. (**C**) Purification of Zika Env:hFc and (**D**) Zika Env:aghFc by Protein A affinity chromatography. The total plant extract and flow-through were subject to Western blotting (left panels), and eluents were stained with Coomassie Brilliant Blue after polyacrylamide gel electrophoresis (right panels). T, total plant extract; FT, flow-through; E, eluents. Bovine serum albumin (BSA) was used as a standard protein for quantitation. Original blots are presented in Supplementary Fig. 1.

The recombinant ZikaEnv:hFc and ZikaEnv:aghFc proteins were expressed in the endoplasmic reticulum (ER) by ER-retention signals (Fig. 1A). After their transient transfection into *N. benthamiana*, we tested the solubility of these proteins in plant extracts. The expression of proteins, such as those in the plant extract (T), and in soluble (S) and insoluble (P) fractions, was verified by Western blotting using an anti-human IgG antibody. The expressed proteins remained in the soluble fraction, with the expression level being higher for ZikaEnv:hFc (Fig. 1B). We purified the ZikaEnv:hFc and ZikaEnv:aghFc proteins for further investigation. After binding to Protein A resin, we confirmed the purified ZikaEnv:hFc and ZikaEnv:aghFc proteins by Coomassie staining. The majority of ZikaEnv:hFc and Zik Env:aghFc proteins were bound to the Protein A resin and exhibited high purity (Fig. 1C,D ).

### Evaluation of the humoral and cellular immune responses afforded by multiple adjuvants

The addition of proper adjuvants is essential for recombinant subunit vaccines, because the vaccine component is easily lysed in the body and inhibits antigen presentation and antibody production. This study examined the humoral and cellular immunity are induced by plant-based (*Nicotiana benthamiana*) recombinant subunit vaccines (ZikaEnv:hFc, ZikaEnv:aghFc) in combination with four adjuvants, as follows. Alum, as a stimulator of the Th2 immune response and antigen presentation, was immunized with monophosphoryl-lipid A (MPL; EcML), which is a TLR4 agonist that induces the Th1 immune response. Moreover, CIA06 as a conjugate of aluminum hydroxide with detoxified LOS (dLOS; EyeGene Inc., Seoul, Republic of Korea), which is another TLR4 agonist, was also used. Finally, CIA09A (EyeGene Inc., Seoul, Republic of Korea) as a conjugate of dLOS and QS-21, which is an immune stimulator, was administered together with the vaccines.

**Figure 2:**
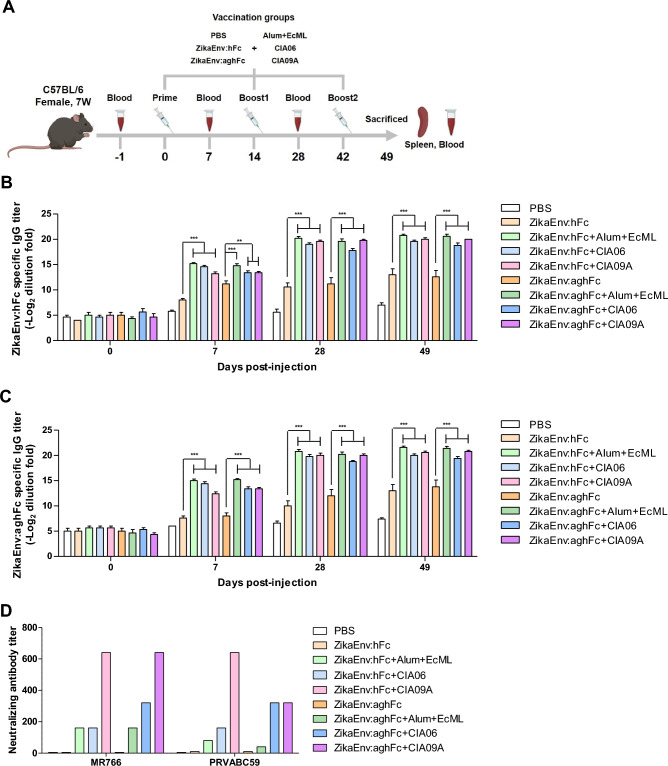
Antibody responses elicited by ZikaEnv:hFc and ZikaEnv:aghFc in the presence of each adjuvant or an adjuvant combination. (**A**) Experimental strategies. C57BL/6 mice (n = 5) were immunized with ZikaEnv:hFc and ZikaEnv:aghFc with each adjuvant or with an adjuvant combination, or with PBS at weeks 0, 2, and 6. The adjuvants used are indicated in all figures. To measure the humoral immune response, blood samples were collected at weeks 0, 1, 4, and 7. (**B**, **C**) An indirect enzyme-linked immunosorbent assay (ELISA) was performed using ZikaEnv:hFc and ZikaEnv:aghFc as coating antigens. Serum samples were diluted at 1:100 and OD 450 values were measured using indirect ELISA. The data are presented as the mean ± SD of five mice per group. Statistical analysis: *P < 0.05, **P < 0.01, ***P < 0.001. (**D**) Virus neutralization titers against ZIKV, as determined using the plaque reduction neutralization test (PRNT) Mouse sera utilized for PRNT measurement were taken 7 weeks post immunization with each adjuvant, an adjuvant combination, or PBS.

One to three doses of the subunit vaccines together with the adjuvants were administered to immunocompetent mice and serum was collected at 7–14 dpi, to examine the levels of Ag-specific antibodies (Fig. 2A). Two subunit vaccines induced antibodies to a certain extent, with each of the adjuvants further increasing the antibody levels. The ZikaEnv:aghFc subunit vaccine induced a higher antibody titer than did the ZikaEnv:hFc vaccine at 7 dpi; however, the responses to these two vaccines were similar at 28 and 49 dpi, with each adjuvant raising a similar amount of Ag-specific antibodies (Fig. 2B,C). Next, we examined the induction of neutralizing antibodies against two ZIKV strains, MR766 and PRVABC59, at 49 dpi. The subunit vaccines alone failed to induce neutralizing antibodies, whereas vaccines administered together with the CIA06 and CIA09A adjuvants induced neutralizing antibodies against the two ZIKV strains at high levels (Fig. 2D). These results indicate that the plant-based subunit vaccines are potent stimulators of humoral immunity when they are administered in conjunction with assorted adjuvants.

**Figure 3:**
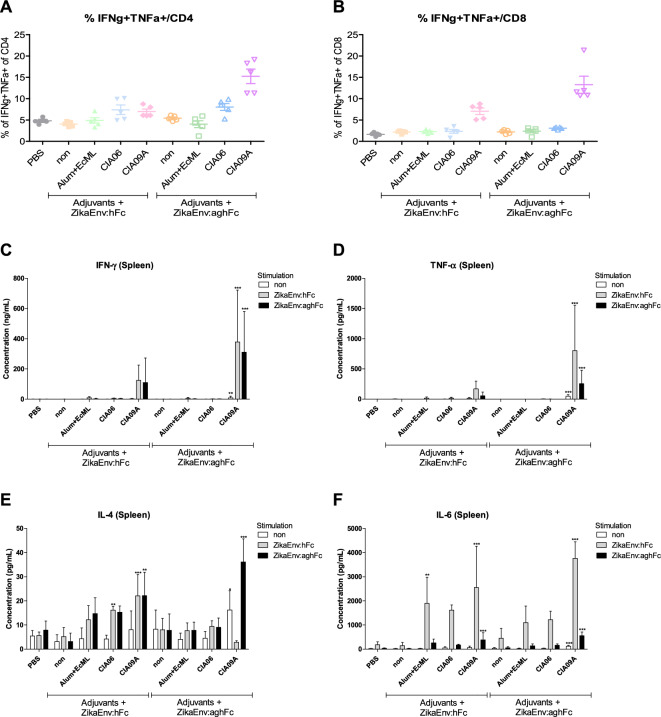
Cellular immune responses elicited by ZikaEnv:hFc and ZikaEnv:aghFc in the presence of each adjuvant or an adjuvant combination. C57BL/6 mice (n = 5) were immunized with ZikaEnv:hFc and ZikaEnv:aghFc with each adjuvant or with an adjuvant combination, or PBS at weeks 0, 2, and 6. The adjuvants used are indicated in all figures. Mice were euthanized at 7 days after the last immunization. To measure the cellular immune response, splenocytes were isolated from five mice per group. (**A**, **B**) Frequencies of IFN-γ- and TNF-α-expressing CD4 + and CD8 + T cells, as determined by flow cytometry. (**C**–**F**) Splenocytes were stimulated with the same dose of (1 μg/mL) of ZikaEnv:hFc, ZikaEnv:aghFc, or PBS. The supernatants were harvested after 48 h of incubation and used to measure the concentrations of IFN-γ, TNF-α, IL-4, and IL-12 using ELISA. The data are presented as the mean ± SD of five mice per group. Statistical analysis: *P < 0.05, **P < 0.01, ***P < 0.001.

Next, we assessed the cellular immunity generated by each vaccine and adjuvant. After three immunizations, we collected splenocytes and examined the intracellular expression of effector cytokines by CD4^+^ and CD8^+^ T cells using flow cytometry. Similar to the humoral response, IFN-γ- and TNF-α-expressing T cells were detected at high levels when the vaccines were administered together with CIA09A (Fig. 3A, B); in contrast, other T-cell cytokines, such as IL-17A, IL-10, and IL-22, were undetectable (data not shown). We further analyzed the secretion of effector cytokines from the splenocytes after restimulation with viral antigens. In line with the results of intracellular staining, IFN-γ and TNF-α were highly secreted in the CIA09A-immunized group (Fig. 3C,D ). Other major T-cell cytokines, such as IL-4 and IL-6, were also well induced by CIA09A (Fig. 3E,F). Here, we observed that the administration of vaccines together with the CIA09A adjuvant induced a potent type I immunity and functioned as a potential stimulator of Th2 and Th17 responses.

### Determination of the optimal doses of vaccines

**Figure 4:**
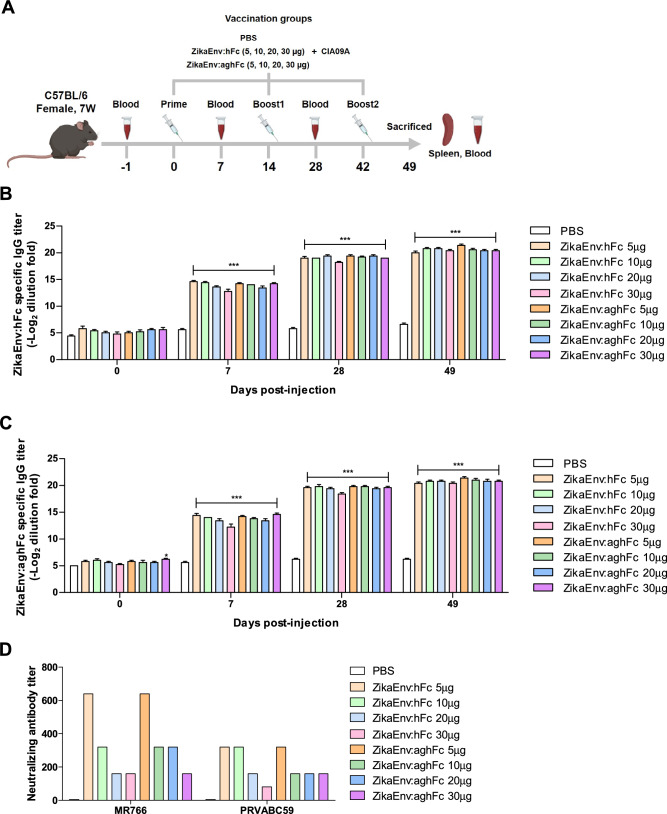
Humoral immune responses elicited according to the amount of Zika Env:hFc and ZikaEnv:aghFc. (**A**) Experimental strategies. C57BL/6 mice (n = 5) were immunized with ZikaEnv:hFc, ZikaEnv:aghFc, or PBS at weeks 0, 2, and 6. To measure the humoral immune response, blood samples were collected at weeks 0, 1, 4, and 7. (**B**, **C**) An indirect enzyme-linked immunosorbent assay (ELISA) was performed using ZikaEnv:hFc and ZikaEnv:aghFc as coating antigens. Serum samples were diluted at 1:100 and OD 450 values were measured using indirect ELISA. The data are presented as the mean ± SD of five mice per group. Statistical analysis: *P < 0.05, **P < 0.01, ***P < 0.001. (**D**) Virus neutralization titers against ZIKV, as determined using the plaque reduction neutralization test (PRNT). Mouse sera utilized for PRNT measurement were taken 7 weeks post immunization with ZikaEnv:hFc, ZikaEnv:aghFc, or PBS.

**Figure 5:**
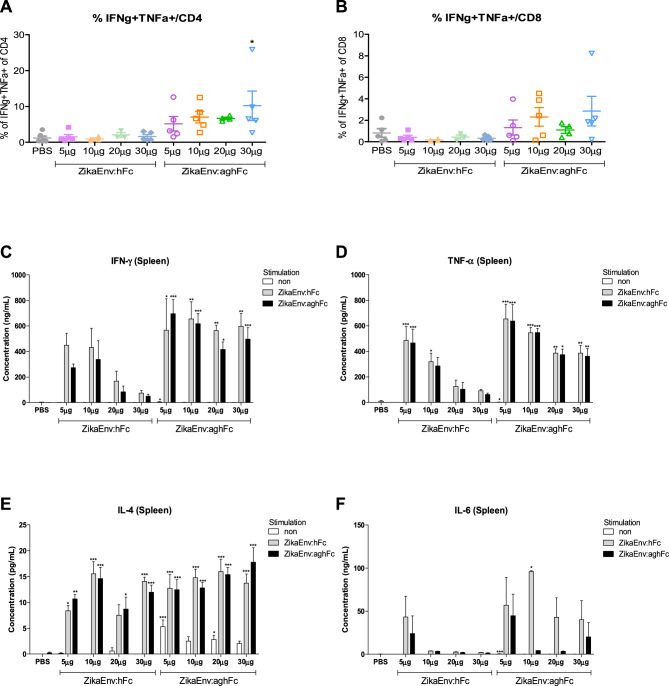
Cellular immune responses elicited according to the amount of Zika Env:hFc and ZikaEnv:aghFc. C57BL/6 mice (n = 5) were immunized with ZikaEnv:hFc, ZikaEnv:aghFc, or PBS at weeks 0, 2, and 6. Mice were euthanized at 7 days after the last immunization. To measure the cellular immune response, splenocytes were isolated from five mice per group. (**A**, **B**) The frequencies of IFN-γ- and TNF-α-expressing CD4^+^ and CD8^+^ T cells are shown. (**C**–**F**) Splenocytes were stimulated with the same dose (1 μg/mL) of ZikaEnv:hFc, ZikaEnv:aghFc, or PBS. The supernatants were harvested after 48 h of incubation and were used to measure the concentrations of IFN-γ, TNF-α, IL-4, and IL-12 using ELISA. The data are presented as the mean ± SD of five mice per group. Statistical analysis: *P < 0.05, **P < 0.01, ***P < 0.001.

In the previous subsection, we confirmed that the two plant-based ZIKV vaccines were efficient regulators of both humoral and cellular immunity when administered together with CIA09A. Next, we performed titration experiments to determine the optimal dose of the vaccines. Immunocompetent mice were immunized 1–3 times with the two vaccine proteins at different doses, i.e., 5, 10, 20, and 30 μg, and serum and splenocytes were collected at 7, 28, and 49 dpi (Fig. 4A). The Ag-specific IgG titers of both ZikaEnv:hFc and ZikaEnv:aghFc were significantly increased by 3–fivefold after vaccine immunizations, and higher titers were maintained after boost immunizations were performed. However, we did not observe a dose-dependent effect of the vaccine antigens (Fig. 4B,C). Next, we examined the neutralizing antibody titer of two ZIKV strains: in the MR766-infected group, 5 μg of the vaccine antigen induced a high neutralizing antibody level, with a similar response being observed in the PRVABC59-infected group; however, higher doses failed to generate additional neutralizing antibodies (Fig. 4D). To evaluate the cellular immunity afforded by our vaccines, splenocytes were collected from immunized mice at 49 dpi. The ZikaEnv:aghFc vaccine stimulated effector T cells that expressed IFN-γ and TNF-α in a dose-dependent manner, especially regarding CD4 + T cells; in contrast, ZikaEnv:hFc injection yielded minimal changes in those effector T-cell populations (Fig. 5A,B). Effector cytokines were further analyzed by ELISA, as follows. Splenocytes from immunized mice were restimulated by each vaccine strain. In line with the results of the intracellular staining, cells from ZikaEnv:aghFc-immunized animals exhibited a strong induction of IFN-γ and TNF-α. Restimulation with 5 μg of the vaccine antigen was most efficient for the induction of type-I cytokine production (Fig. 5C,D). We further examined IL-4 and IL-6 secretion and evaluated the potential induction of Th2 and Th17 by the vaccines. IL-4 was fairly well induced by each vaccine dose, whereas IL-6 induction varied (Fig. 5E,F). Here, we showed that 5 μg of the vaccine antigen was sufficient to induce both humoral and cellular immunity, which suggests that ZikaEnv:aghFc is a better candidate for the induction of cellular immunity.

### ZikaEnv:aghFc induced protective immunity against ZIKV and DENV in neonatal mice

**Figure 6:**
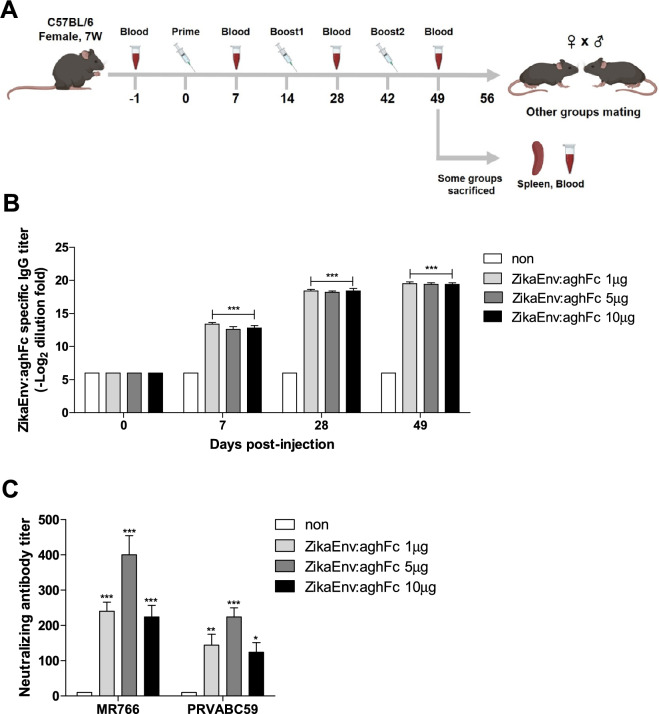
Humoral immune responses elicited by ZikaEnv:aghFc. (**A**) Experimental strategies. C57BL/6 mice (n = 5) were immunized with ZikaEnv:aghFc or PBS at weeks 0, 2, and 6. To measure the humoral immune response, blood samples were collected at weeks 0, 1, 4, and 7. (**B**) An indirect enzyme-linked immunosorbent assay (ELISA) was performed using ZikaEnv:aghFc as a coating antigen. Serum samples were diluted at 1:100 and OD 450 values were measured using indirect ELISA. The data are presented as the mean ± SD of five mice per group. Statistical analysis: *P < 0.05, **P < 0.01, ***P < 0.001. (**C**) Virus neutralization titers against ZIKV, as determined using the plaque reduction neutralization test (PRNT). Mouse sera utilized for PRNT measurement were taken 7 weeks post immunization with ZikaEnv:aghFc or PBS.

**Figure 7:**
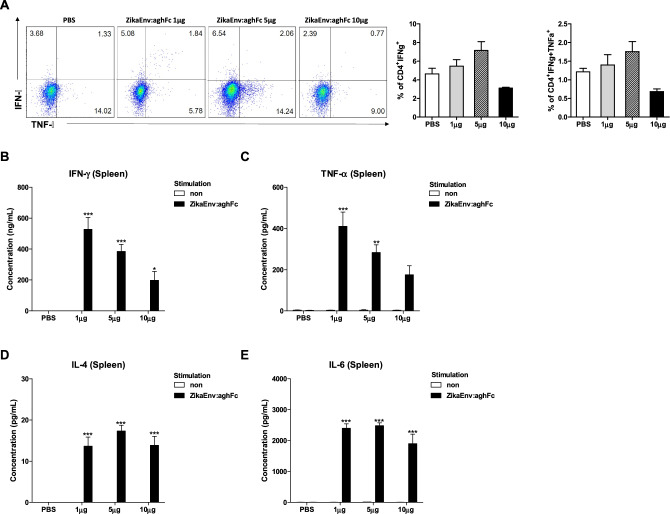
Cellular immune responses elicited by ZikaEnv:aghFc. C57BL/6 mice (n = 5) were immunized with ZikaEnv:aghFc or PBS at weeks 0, 2, and 6. Mice were euthanized at 7 days after the last immunization. To measure the cellular immune response, splenocytes were isolated from five mice per group. (**A**) Frequencies of IFN-γ- and TNF-α-expressing CD4^+^ and CD8^+^ T cells. (**B**–**E**) Splenocytes were stimulated with the same dose (1 μg/ml) of ZikaEnv:aghFc or PBS. The supernatants were harvested after 48 h of incubation and used to measure the concentrations of IFN-γ, TNF-α, IL-4, and IL-12 using ELISA. The data are presented as the mean ± SD of five mice per group. Statistical analysis: *P < 0.05, **P < 0.01, ***P < 0.001.

As the ZikaEnv:aghFc vaccine was shown to be a dynamic immune modulator, we wondered whether it induces vertically transferred immunity. First, we examined the vaccine-mediated humoral immunity in female mice treated with 1, 5, and 10 μg of the vaccine antigen three times, on days 0, 14, and 42 (Fig. 6A). We found that 1 μg of the vaccine and two vaccine doses were sufficient to generate a significant level of antigen-specific antibodies (Fig. 6B). When the female mice were challenged with two ZIKV strains, 5 μg of vaccine generated the highest level of neutralizing antibodies, with the antibody titer being higher in mice infected with the MR766 vs. the PRVABC53 strain (Fig. 6C). To evaluate cellular immunity, we measured effector cytokines from lymphocytes. Similar to humoral immunity, 1 and 5 μg of the vaccine triggered IFN-γ- and TNF-α-expressing CD4^+^ T cells (Fig. 7A). The restimulation of splenocytes with the vaccine antigen led to the secretion of a higher level of IFN-γ and TNF-α in cells at lower vaccine doses (Fig. 7B–E).

**Figure 8:**
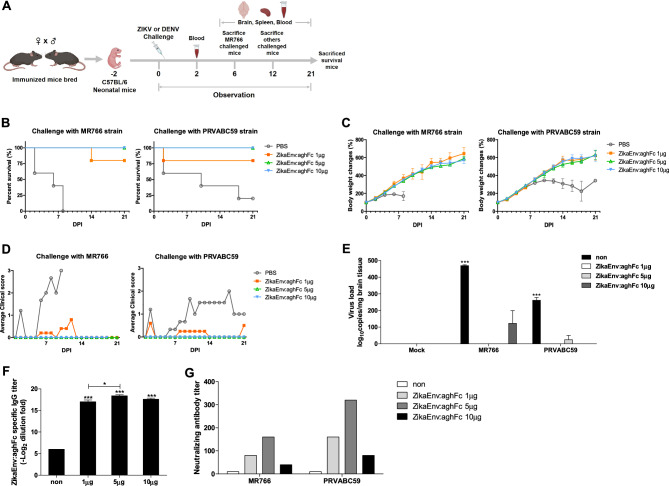
Assessment of the protection against ZIKV challenge afforded by transfer of ZikaEnv:aghFc-specific Ab. (**A**) Experimental strategies. C57BL/6 mice (n = 5) were immunized with ZikaEnv:aghFc or PBS at weeks 0, 2, and 6. Immunized mice were bred as homozygous breeding pairs. (**B**–**D**) The survival rate, body weight, and clinical score of C57BL/6 neonatal mice (n = 5 per group) were monitored up to 3 weeks post-infection. Two-day-old neonatal mice were inoculated with 10^6^ TCID_50_/mouse for the MR766 strain (**A**–**C**) or 10^6^ TCID_50_/mouse for the PRVABC59 strain. (**B**) The survival rate is presented using Kaplan–Meier survival curves. (**C**) The body weight data are represented as the mean ± SD of five mice per group. (**D**) Mice were allocated a clinical score (range, 0–3) based on the most severe clinical sign observed, as follows: normal appearance (0); staggering walk, wide stance, or paralysis of the hind legs (1); 25% weight loss or labored breathing (2); and death (3). (**E**) Two-day-old neonatal mice were inoculated with 10^6^ TCID_50_/mouse for the MR766 strain or 10^6^ TCID_50_/mouse for the PRVABC59 strain. After 6 days for the MR766 ZIKV strain or after 12 days for the PRVABC59 ZIKV strain challenge, ZIKV RNA levels in the mouse brain were measured by qRT-PCR. The data are presented as the mean ± SD of five mice per group. (**F**) To measure ZikaEnv:aghFc-specific IgG antibodies, blood samples were collected on day 2. Serum samples were diluted at 1:100 and OD 450 values were measured using indirect ELISA. The data are presented as the mean ± SD of five mice per group. Statistical analysis: *P < 0.05, **P < 0.01, ***P < 0.001. (**G**) Virus neutralization titers against ZIKV, as determined using the plaque reduction neutralization test (PRNT). To measure PRNT, blood samples were collected on day 2.

Finally, we examined whether the vaccine afforded vertically transferred immunity. ZikaEnv:aghFc-immunized males and females were bred and neonates were challenged with two ZIKV strains (MR766 and PRVABC59) at 10^6^ TCID_50_/animal (Fig. 8A). In both cases, immunization with 1 and 5 μg of ZikaEnv:aghFc protected 80% and 100% of the pups against ZIKV, respectively, and immunized mice constantly gained body weight (Fig. 8B,C). ZIKV infection leads to the development of microcephaly in neonates by targeting neural progenitor cells that are associated with neural system development^43^. We examined clinical signs in pups, such as staggering march, wide stance, paralysis of the hind legs, and labored breathing^44^. Although a vaccine dose of 1 μg suppressed clinical symptoms to some extent, 5 and 10 μg of ZikaEnv:aghFc thoroughly protected the mice against neurological disorder (Fig. 8D). Moreover, the higher doses of the vaccine efficiently protected the brain tissues against viral invasion (Fig. 8E). In those protected animals, the ZikaEnv:aghFc-specific IgG antibody titer and neutralizing antibodies were significantly increased in the serum at 2 dpi (Fig. 8F,G). Collectively, these data indicate that the vaccine-generated immunity was vertically transferred and constituted a potent protective immunity in the next generation.

**Figure 9:**
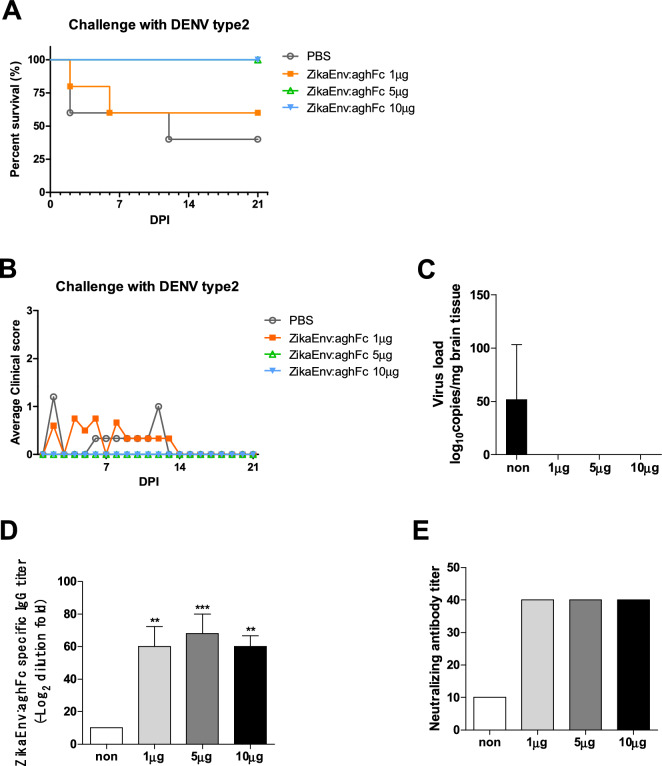
Assessment of the protection against DENV challenge afforded by the transfer of ZikaEnv:aghFc-specific Ab. C57BL/6 mice (n = 5) were immunized with ZikaEnv:aghFc or PBS at weeks 0, 2, and 6. Immunized mice were bred as homozygous breeding pairs. (**A**, **B**) The survival rate and clinical score of C57BL/6 neonatal mice (n = 5 per group) were monitored up to 3 weeks post-infection. Two-day-old neonatal mice were inoculated with DENV type 2 at 10^6^ TCID_50_/mouse. (**A**) The survival rate is presented using Kaplan–Meier survival curves. (**B**) Mice were allocated a clinical score (range, 0–3) based on the most severe clinical sign observed, as follows: normal appearance (0); staggering walk, wide stance, or paralysis of the hind legs (1); 25% weight loss or labored breathing (2); and death (3). (**C**) After 12 days of DENV type 2 challenge, DENV RNA levels in the mouse brain were measured by qRT-PCR. (**D**) To measure ZikaEnv:aghFc-specific IgG antibodies, blood samples were collected on day 2. Serum samples were diluted at 1:100 and OD 450 values were measured using indirect ELISA. (**E**) Virus neutralization titers against ZIKV, as determined using the plaque reduction neutralization test (PRNT). To measure PRNT, blood samples were collected on day 2. The data are presented as the mean ± SD of five mice per group. Statistical analysis: *P < 0.05, **P < 0.01, ***P < 0.001.

The E protein in DENV has a 54%–59% amino-acid identity with that of ZIKV^45^. Because of genetic similarity, ZIKV infection generates cross-reactive antibodies that target the highly conserved DII-FL epitope in the E protein, which can modulate antibody-dependent enhancement (ADE)^46^. Here, we investigated whether cross-protection against DENV was afforded by ZikaEnv:aghFc immunization, and whether this was vertically transferred. Higher vaccine doses (5 and 10 μg) protected pups against a DENV type 2 challenge (Fig. 9A). Moreover, the clinical signs were ameliorated and DENV invasion into brain tissues was inhibited by the immunization with ZikaEnv:aghFc (Fig. 9B,C). Importantly, the vertically transferred antibody titers for both vaccine-antigen-specific IgG and neutralizing antibodies were significantly increased by the vaccination (Fig. 9D,E). These data demonstrate that the ZikaEnv:aghFc vaccine induced cross-protection against DENV infection.

## Discussion

ZIKV rapidly spread to South and Central America after it was reported in Brazil in 2015. Protection against ZIKV is essential in women during pregnancy, a period of vulnerability to congenital Zika syndrome, which induces fatal malformation in neonates^47^. Researchers have dedicated great efforts toward the development of ZIKV vaccines; as a result, several vaccine candidates are under phase I trials and two candidates are under phase II clinical trials^12^. In these trials, purified-inactivated, live-attenuated, DNA, mRNA, and recombinant vaccines were used, whereas attenuated vaccines were excluded because of safety issues^12^.

There are concerns that *N*-glycans as plant-produced glycoproteins can act as allergens. In fact, the *N*-glycans of Api g 5, which is a glycoprotein allergen from celery, bind to immunoglobulin E in vitro^48^. The presence of α (1,3)-fucose and β (1,2)-xylose on *N*-glycans from plants is the basis of the high degree of cross-reactivity that is associated with carbohydrate-specific IgE antibodies^49–51^. However, a recent study reported that a vaccine against seasonal influenza produced from *N. benthamiana* by Medicago Inc. elicited a transient IgG or IgE response, although it was not associated with allergy or hypersensitivity symptoms^52^. Here, to minimize the adverse immune responses triggered by plant-specific *N*-glycans, we removed the *N*-glycan from the Fc fragment.

The ZIKV E protein plays pivotal roles in viral assembly, attachment, entry, and fusion^10,11^; moreover, it comprises epitopes that induce neutralizing and protective antibodies^12^. This study developed plant-based ZIKV recombinant subunit vaccines (ZikaEnv:hFc and ZikaEnv:aghFc) that targeted the ZIKV E protein and were generated from *N. benthamiana* plants via transient expression. Subsequently, we examined the vaccine-induced protective immunity against fatal viral infection challenges.

Recombinant subunit vaccines are known to be safer than live attenuated vaccines. Concurrently, the selection of optimal adjuvants is critical because of the lower immunogenicity of the vaccine^11^. Here, we examined the immunogenicity of several adjuvant candidates, i.e., Alum, EcML (MPL), CIA06, and CIA09A. The addition of each of these adjuvants to the vaccines (ZikaEnv:hFc and ZikaEnv:aghFc) significantly increased the levels of Ag-specific IgG and neutralizing antibodies. Of note, CIA09A addition induced the highest levels of both humoral and cellular immunities, such as antibody titers and the generation of CD4^+^ and CD8^+^ T cells and their effector cytokines, IFN-γ, TNF-α, IL-4, and IL-6. This was in line with the results of a previous study that reported potent immunogenicity against gE and VZV when the vaccine and adjuvant were stored in a co-lyophilized state^42^. This implies that our ZIKV vaccine can be co-lyophilized with CIA09A and stored in the same container.

We found that a lower vaccine dose afforded the optimal amount of antigen, which triggered higher antibody titers and effector cytokine production. Moreover, ZikaEnv:aghFc induced a stronger immunogenicity than did ZikaEnv:hFc. In the ZikaEnv:aghFc vaccine, the glycosylation region of the Fc domain of human immunoglobulin was removed, to prevent unexpected immune responses to plant- originated glucose residues.

To investigate whether the ZikaEnv:aghFc vaccine protects hosts (adult females) from ZIKV infection, we examined the potent humoral and cellular immunity induced against two ZIKV strains (MR766 and PRVABC59). ZIKV infection during pregnancy allows this virus to invade multiple regions of the CNS tissues of the fetuses, including the cerebrum, where it then expands^40^ and inhibits neuronal stem cell (NSC) proliferation, thus leading to neuronal disorders^53,54^. Therefore, we assessed whether the immunity afforded by the ZikaEnv:aghFc vaccine was vertically transferred. Pups from three-time-immunized parents exhibited high antibody titers and survived after lethal viral infections. Moreover, pups from vaccinated parents showed little viral spread in the neonatal brain and diminished clinical symptoms.

ZIKV and DENV are flaviviruses that share genetic similarity and are highly contagious because they are transmitted by *Aedes* mosquito vectors^45,55^. Because of the genetic closeness between the E proteins of ZIKV and DENV, ZIKV vaccination can induce cross-reactive antibodies that target the highly conserved DII-FL epitope and result in antibody-dependent enhancement (ADE)^46^. Therefore, a ZIKV vaccine should avoid the potential ADE caused by exposure to DENV^12^. The vertically transferred immunity afforded by ZikaEnv:aghFc immunization exhibited valid cross-protection and a high level of neutralizing antibodies to DENV. This constitutes indirect evidence that our vaccine avoids the potential risk of ADE.

In summary, this study showed that our plant-based recombinant subunit ZIKV vaccine afforded a substantial level of protective immunity. Of note, this immunity was vertically transferred and protected the next generation of mice against ZIKV infection. In a previous study, we developed a ZIKV E protein subunit vaccine that conferred partial protection against ZIKV and DENV^15^. The vaccine produced in the current study afforded improved protection against both ZIKV and DENV, which indicates that plant-based vaccine production is potentially applicable in advanced studies of nonhuman primates and clinical trials.

## Methods

### Cells and viruses

Vero cells (KCLB, Republic of Korea) were cultured in minimal essential medium α (MEM-α) (Gibco, USA) supplemented with 10% fetal bovine serum (FBS; Gibco, USA), and Vero 76 cells (ATCC, USA) were grown in Dulbecco’s Minimal Essential Medium (DMEM; Gibco, USA) containing 10% FBS at 37 °C in 5% CO_2_ until they formed monolayers. BEI Resources provided the Asian-lineage PRVABC59 ZIKV strain (BEI Resources No. NR-50240) and the African-lineage MR766 ZIKV strain (BEI Resources No. NR-50065). Vero76 cells were used to propagate the MR766 strain, and Vero cells were used to proliferate the PRVABC59 strain, both at an MOI of 0.01. Plaque assays were used to measure ZIKV stock titers of both cell lines, which were then stored at –80 °C.

### Cloning of the Zika Env:hFc and Zika Env:aghFc genes

The sequence of the gene encoding the Zika virus envelope protein (Zika Env) was obtained from the National Center for Biotechnology Information (NCBI) database (GenBank Accession Number; MW680970.1) and optimized for expression in *Nicotiana benthamiana* (https://zendto.bioneer.co.kr/codon/index.py), prior to gene synthesis. For Zika Env:hFc expression, genes encoding the signal peptide of the chaperone-binding protein (NB), Zika Env, constant fragment of human immunoglobulin G (hFc), and endoplasmic reticulum (ER)-retention signal were fused sequentially, then cloned into a pTEX vector harboring a MacT promoter and an RD29B terminator. For Zika Env:aghFc expression, a peptide linker (L) was substituted with upper hinge region of hFc, and the asparagine (N) residue of the only *N*-glycosylation site of hFc was replaced with alanine (A). The nucleotide sequence was verified by sequencing (Bioneer, Korea).

### Transient expression of recombinant Zika Env:hFc and Zika Env:aghFc

Plasmids for the expression of recombinant Zika Env:hFc and Zika Env:aghFc were transformed into the *Agrobacterium tumefaciens* GV3101 strain (Lifeasble, Australia) by electroporation. Transformed *A. tumefaciens* cells were grown for 16 h in 5 mL of yeast extract peptone (YEP) liquid medium supplemented with 50 mg/L kanamycin and 25 mg/L rifampicin. Next, 1 mL of cultured recombinant agrobacteria was inoculated into 1 L of fresh YEP medium and cultured for a further 16 h at 28 °C. Recombinant agrobacteria were then pelleted by centrifugation at 7341 × *g* for 5 min at 4 °C and resuspended at the desired concentration (as determined by measuring OD 600) in a solution consisting of 10 mM 2-(*N*-morpholino) ethane sulfonic acid (MES) (Duksan, Korea), 10 mM magnesium chloride (Sigma-Aldrich, USA), and 100 mM acetosyringone (Sigma-Aldrich, USA) at pH 5.6. Agroinfiltration was carried out using vacuum. After 4 days, the leaves were harvested for protein purification.

### Purification of the Zika Env:hFc and Zika Env:aghFc proteins

The harvested leaves were then crushed and incubated for 30 min in an extraction buffer consisting of 50 mM sodium phosphate (pH 8.0), 300 mM sodium chloride, 0.5% Triton X-100, 100 mM sodium sulfite, 1.5% polyvinylpolypyrrolidone (PVPP), and 1 mM phenylmethylsulfonylfluoride (PMSF). After centrifugation, small amounts of the supernatant and pellet fractions were collected and subjected to Western blot analysis for testing solubility using an HRP-conjugated anti-human IgG Fc antibody (Fortis Life Science, USA). The supernatant was then mixed with Puriose ProA 90E Resin (Puriogen, Korea) for 1 h at 4 °C, and the bound proteins were eluted with 100 mM sodium citrate (pH 3.0). Finally, the pH of the eluate was adjusted to 7.3 with 1.5 M Tris–Cl (pH 8.8), and the purified proteins were assessed and quantified by sodium dodecyl sulfate polyacrylamide gel electrophoresis (SDS–PAGE) by comparing with bovine serum albumin.

### Mice

C57BL/6 mice were purchased from Doo Yeol Biotech (Seoul, Korea). Mice were housed at the Animal Laboratory Center of Kangwon National University with free access to food and water and a 12-h light–dark cycle. Mice were immunized intramuscularly (i.m.) using the recombinant ZIKV E protein (Zika Env:hFc, ZikaEnv:aghFc) mentioned above in the presence or absence of various adjuvants: Alum (i.e., aluminum hydroxide, 500 μg/mouse; InvivoGen, San Diego, CA, USA), EcML (i.e., *Escherichia coli*-produced monophosphoryl lipid A, 20 μg/mouse; Eubiologics, Chuncheon-si, Korea), CIA06 (50 μL/mouse; EyeGene Inc., Seoul, Republic of Korea), and CIA09A (50 μL/mouse; EyeGene Inc., Seoul, Republic of Korea). Mice were inoculated three times, at 0, 14, and 42 days. For the study of cellular immune responses, the mice were euthanized with CO_2_ and splenocytes were harvested at 7 days post-last dose (dpi 49). Serum was collected from the mice at 0, 14, and 49 days. After the experiment was completed, each group that had received the vaccination was mated with homozygous breeding pairs. Two-day-old C57BL/6 mice were subcutaneously injected with phosphate-buffered saline (PBS), as a negative control; with each of the ZIKV strains (MR766, PRVABC59); or with DENV (Type 2), at 1 × 10^6^ TCID_50_/mouse. For the challenge test, we used two ZIKV strains (MR766 and PRVABC59) whose pathogenicity was confirmed in previous studies as challenge viruses^56^. Suckling mice were observed up to 21 days after the challenge, to assess survival rate, weight loss, and aberrant behavior. Based on the most severe clinical symptom that was observed, the animals received a score (range, 0–3), as follows^44^: normal appearance (0); staggering walk, wide stance, or paralysis of the hind leg (1); 25% weight loss or labored breathing (2); and death (3). The mouse sera were collected at 0 and 2 dpi. After 6 days for the MR766 ZIKV strain or after 12 days for the PRVABC59 ZIKV strain challenge, some mice were euthanized with CO_2_ and their brains were collected. Based on the findings of prior studies, the sample collection time after each virus challenges was chosen taking into account the LD_50_ established^15,56,57^. Except for the group used for survival rate study, all mice were euthanized with CO_2_ after the test. This work was carried out in compliance with the ARRIVE guidelines and approved by the Institutional Animal Care and Use Committee (IACUC) of Kangwon National University (No: KW-210209–1, KW-210528–1, KW-210707–2, KW-220112–2). Pre-defined animal welfare endpoints were also approved by the Institutional Animal Care and Use Committee (IACUC) of Kangwon National University. All experiments were performed in accordance with relevant guidelines and regulations.

### Enzyme-linked immunosorbent assay (ELISA)

Recombinant ZIKV E protein (Zika Env:hFc, ZikaEnv:aghFc)-specific IgG antibodies were detected by indirect enzyme-linked immunosorbent assay (ELISA). Each well of a 96-well microtiter plate (Nunc-Immuno Plates; Thermo Scientific, UK) was coated with 100 ng of ZikaEnv:hFc or ZikaEnv:aghFc at 4 °C overnight. Each well was then washed three times with 0.05% Tween 20 in PBS (PBS-T) before being blocked for 2 h at 37 °C with 1% bovine serum albumin (BSA; Millipore, USA) in PBS-T. The plates were washed three times with PBS-T. After their dilution at a ratio of 1:100 with 0.1% BSA in PBS-T, 100 μL of the sera was added to each well for 2 h at 37 °C. After three washes, the plates were incubated with an HRP-conjugated goat anti-mouse IgG heavy and light chain antibody (1:10,000; Bethyl Laboratories, USA) at 37 °C for 1 h, then washed with PBS-T. The reaction was detected by the tetramethylbenzidine (TMB) substrate (Surmodics, USA) in the dark and stopped using 2 N H_2_SO_4_. The optical density of the plates was read at 450 nm in an ELISA plate reader (BioTek, Winooski, NT, USA. Splenocytes (5 × 10^5^ cells/well) were cultured in 96-well cell culture plates (SPL, Korea) and stimulated with Zika Env:hFc or ZikaEnv:aghFc (100 ng/well) for 48 h. Subsequently, the media from the splenocyte cultures were collected and cytokine analysis was carried out using ELISA with the ELISA MAX (Deluxe Set) mouse IFN-γ, TNF-α, IL-4, and IL-6 ELISA kits (BioLegend, CA, USA), according to the manufacturer’s protocol.

### Plaque reduction neutralization test

Vero or Vero 76 cells were grown overnight in DMEM containing 10% FBS after being seeded in a 24-well plate at a density of 1 × 10^5^ cells/well. Sera were serially diluted in DMEM after being heat inactivated at 56 °C for 30 min. ZIKV was prepared by diluting it to 2 × 10^2^ PFU/ml in DMEM, mixing it with an equal volume of diluted serum samples, and then incubating the mixture for 30 min at 37 °C. The mixture was used to infect Vero or Vero 76 cells after an incubation period of 2 h at 37 °C. After washing, the cells were incubated at 37 °C for 5 or 14 days in full DMEM containing 1.4% methyl cellulose or 1% sea plaque agar. The cells were fixed in 4% paraformaldehyde and then stained with 0.1% crystal violet dye. The percentage inhibition of virus infectivity was estimated by comparing the plaque counts of immune sera with those of the serum-free control.

### Flow cytometry analysis

IFN-γ- and/or TNF-α-expressing cells among CD4^+^ and CD8^+^ T cells were assessed by flow cytometry. At 49 dpi, spleens from each group were collected. After RBC lysis, the surfaces of splenocytes were stained with antibodies against mouse CD3 (Clone: 17A2, BioLegend, CA, USA), CD4 (Clone: RM4-5, BioLegend, CA, USA), and CD8 (Clone: 53–6.7, BioLegend, CA, USA). After intracellular fixation and permeabilization using a buffer set (Thermo Scientific, USA), antibodies against mouse IFN-γ (XMG1.2, BioLegend, CA, USA) and TNF-α (XMG1.2, BioLegend, CA, USA) were applied according to the manufacturer’s manual.

### Supplementary Information

Supplementary Information.

## Data Availability

All the data generated during the current study are included in the manuscript.
