# Supplementary material for: Plant-expressed Zika virus envelope protein elicited protective immunity against the Zika virus in immunocompetent mice
Source: Sci Rep. 2023 Dec 27;13:22955. doi: 10.1038/s41598-023-47428-7 (PMC10752873; doi:10.1038/s41598-023-47428-7)
Supplement: Supplementary file 1 — Supplementary Information. [file 41598_2023_47428_MOESM1_ESM.docx]

**Supplementary Figures**


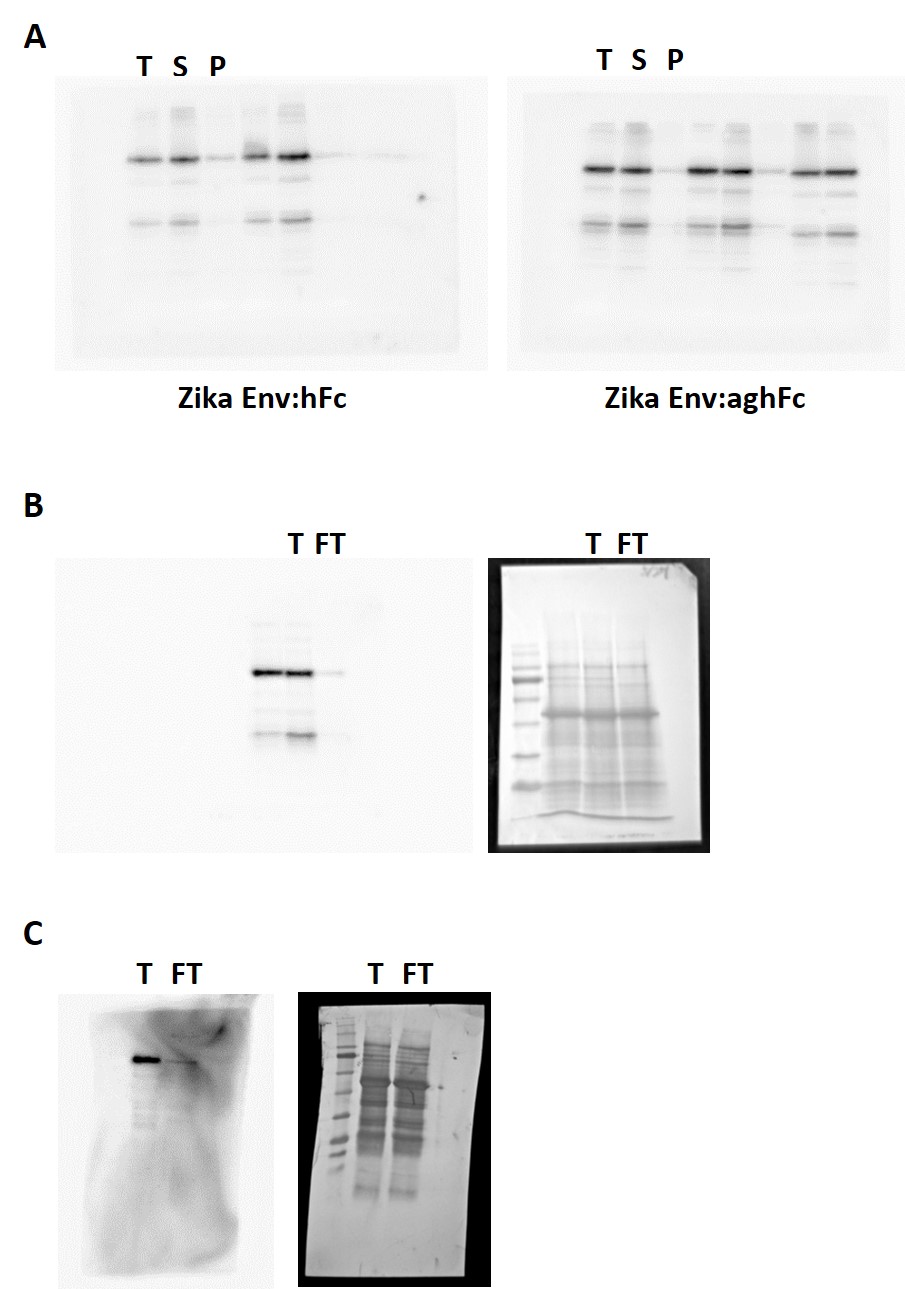


**Supplementary Figure 1. Expression and purification of Zika Env:hFc and Zika Env:aghFc.** (A) Expression and solubility of recombinant Zika Env:hFc and Zika Env:aghFc in *Nicotiana benthamiana*. Each fraction was subjected to Western blot analysis using an HRP-conjugated anti-human IgG antibody. T, plant total extract; S, soluble fraction; P, pellet fraction. (B) Purification of Zika Env:hFc and (C) Zika Env:aghFc by Protein A affinity chromatography. The total plant extract and flow-through were subject to Western blotting. T, total plant extract; FT, flow-through. **Supplementary Methods**

**Supplementary Methods 1. Amino acid sequences of ZikaEnv:hFc and ZikaEnv:aghFc**

(A) ZikaEnv:hFc

GSIRCIGVSNRDFVEGMSGGTWVDVVLEHGGCVTVMAQDKPTVDIELVTTTVSNMAEVRSYCYEASISDMASDSRCPTQGEAYLDKQSDTQYVCKRTLVDRGWGNGCGLFGKGSLVTCAKFACSKKMTGKSIQPENLEYRIMLSVHGSQHSGMIVNDTGHETDENRAKVEITPNSPRAEATLGGFGSLGLDCEPRTGLDFSDLYYLTMNNKHWLVHKEWFHDIPLPWHAGADTGTPHWNNKEALVEFKDAHAKRQTVVVLGSQEGAVHTALAGALEAEMDGAKGRLSSGHLKCRLKMDKLRLKGVSYSLCTAAFTFTKIPAETLHGTVTVEVQYAGTDGPCKVPAQMAVDMQTLTPVGRLITANPVITESTENSKMMLELDPPFGDSYIVIGVGEKKITHHWHRSGSTIGKAFEATVRGARRMAVLGDTAWDFGSVGGALNSLGKGIHQIFGAAFKAREPKSCDKTHTCPPCPAPELLGGPSVFLFPPKPKDTLMISRTPEVTCVVVDVSHEDPEVKFNWYVDGVEVHNAKTKPREEQYNSTYRVVSVLTVLHQDWLNGKEYKCKVSNKALPAPIEKTISKAKGQPREPQVYTLPPSREEMTKNQVSLTCLVKGFYPSDIAVEWESNGQPENNYKTTPPVLDSDGSFFLYSKLTVDKSRWQQGNVFSCSVMHEALHNHYTQKSLSLSPGKHDEL

(B) ZikaEnv:aghFc

GSIRCIGVSNRDFVEGMSGGTWVDVVLEHGGCVTVMAQDKPTVDIELVTTTVSNMAEVRSYCYEASISDMASDSRCPTQGEAYLDKQSDTQYVCKRTLVDRGWGNGCGLFGKGSLVTCAKFACSKKMTGKSIQPENLEYRIMLSVHGSQHSGMIVNDTGHETDENRAKVEITPNSPRAEATLGGFGSLGLDCEPRTGLDFSDLYYLTMNNKHWLVHKEWFHDIPLPWHAGADTGTPHWNNKEALVEFKDAHAKRQTVVVLGSQEGAVHTALAGALEAEMDGAKGRLSSGHLKCRLKMDKLRLKGVSYSLCTAAFTFTKIPAETLHGTVTVEVQYAGTDGPCKVPAQMAVDMQTLTPVGRLITANPVITESTENSKMMLELDPPFGDSYIVIGVGEKKITHHWHRSGSTIGKAFEATVRGARRMAVLGDTAWDFGSVGGALNSLGKGIHQIFGAAFKARGGGGSGGGGSPPCPAPELLGGPSVFLFPPKPKDTLMISRTPEVTCVVVDVSHEDPEVKFNWYVDGVEVHNAKTKPREEQYASTYRVVSVLTVLHQDWLNGKEYKCKVSNKALPAPIEKTISKAKGQPREPQVYTLPPSRDELTKNQVSLTCLVKGFYPSDIAVEWESNGQPENNYKTTPPVLDSDGSFFLYSKLTVDKSRWQQGNVFSCSVMHEALHNHYTQKSLSLSPGKDEL

**Supplementary Methods 2. Nucleotide sequences of ZikaEnv:hFc and ZikaEnv:aghFc**

(A) ZikaEnv:hFc

ATGGCTCGCTCGTTTGGAGCTAACAGTACCGTTGTGTTGGCGATCATCTTCTTCGGTGAGTGATTTTCCGATCTTCTTCTCCGATTTAGATCTCCTCTACATTGTTGCTTAATCTCAGAACCTTTTTTCGTTGTTCCTGGATCTGAATGTGTTTGTTTGCAATTTCACGATCTTAAAAGGTTAGATCTCGATTGGTATTGACGATTGGAATCTTTACGATTTCAGGATGTTTATTTGCGTTGTCCTCTGCAGGATCCATACGATGTATTGGTGTTTCAAATAGGGATTTTGTTGAAGGCATGAGTGGAGGGACATGGGTGGACGTTGTATTGGAGCACGGCGGTTGCGTAACCGTGATGGCCCAAGATAAGCCAACTGTTGACATTGAACTGGTCACGACTACTGTGTCAAATATGGCTGAAGTGCGGAGCTATTGTTATGAAGCATCTATTAGTGATATGGCTAGTGATTCAAGATGTCCTACACAGGGAGAAGCATATCTGGATAAGCAGTCCGATACACAATATGTATGCAAACGTACTCTTGTTGATAGGGGTTGGGGTAACGGTTGCGGATTATTCGGCAAGGGTTCCTTGGTTACTTGTGCTAAATTCGCGTGTAGCAAAAAGATGACAGGTAAGTCGATACAGCCTGAGAATCTTGAGTACCGCATCATGTTGTCTGTTCATGGTAGTCAACATTCTGGGATGATCGTCAATGACACTGGGCATGAGACTGATGAAAACAGAGCAAAGGTTGAGATTACTCCCAATTCACCAAGGGCTGAAGCTACTCTCGGAGGATTTGGTTCTTTAGGATTGGATTGCGAGCCGAGAACTGGACTAGATTTTTCTGATCTTTACTACCTTACTATGAATAACAAACATTGGCTCGTTCACAAAGAATGGTTTCACGACATTCCACTTCCTTGGCATGCCGGAGCAGACACTGGTACTCCTCATTGGAATAACAAAGAGGCTCTGGTCGAATTTAAGGACGCTCACGCAAAAAGACAAACAGTTGTGGTTTTAGGTAGCCAGGAGGGGGCAGTTCATACGGCTTTGGCAGGTGCCTTGGAAGCTGAAATGGATGGTGCCAAGGGTCGATTGTCCAGTGGTCACTTGAAGTGTAGATTGAAGATGGATAAACTTCGTCTTAAGGGGGTGTCATATTCTCTGTGCACTGCTGCTTTTACTTTCACAAAAATACCGGCAGAAACGTTACATGGTACCGTGACAGTCGAGGTTCAATATGCTGGAACTGATGGGCCTTGTAAGGTACCAGCCCAGATGGCTGTCGATATGCAAACCTTAACACCAGTTGGAAGACTTATAACTGCTAATCCCGTTATTACAGAGTCTACTGAAAATTCTAAAATGATGCTAGAGCTTGATCCTCCATTTGGAGACTCTTACATTGTGATTGGAGTTGGTGAAAAAAAGATTACTCATCATTGGCATAGGTCTGGTAGTACAATCGGTAAAGCATTTGAAGCTACCGTAAGGGGCGCGAGAAGAATGGCTGTTCTTGGAGATACAGCTTGGGATTTTGGATCAGTAGGTGGAGCCCTCAACTCACTCGGAAAAGGTATTCACCAGATCTTCGGTGCAGCATTCAAGGCCCGGGAGCCCAAATCTTGTGACAAAACTCACACATGCCCACCGTGCCCAGCACCTGAACTCCTGGGGGGACCGTCAGTCTTCCTCTTCCCCCCAAAACCCAAGGACACCCTCATGATCTCCCGGACCCCTGAGGTCACATGCGTGGTGGTGGACGTGAGCCACGAAGACCCTGAGGTCAAGTTCAACTGGTACGTGGACGGCGTGGAGGTGCATAATGCCAAGACAAAGCCGCGGGAGGAGCAGTACAACAGCACGTACCGTGTGGTCAGCGTCCTCACCGTCCTGCACCAGGACTGGCTGAATGGCAAGGAGTACAAGTGCAAGGTCTCCAACAAAGCCCTCCCAGCCCCCATCGAGAAAACCATCTCCAAAGCCAAAGGGCAGCCCCGAGAACCACAGGTGTACACCCTGCCCCCATCCCGTGAGGAGATGACCAAGAACCAGGTCAGCCTGACCTGCCTGGTCAAAGGCTTCTATCCCAGCGACATCGCCGTGGAGTGGGAGAGCAATGGGCAGCCGGAGAACAACTACAAGACCACGCCTCCCGTGCTGGACTCCGACGGCTCCTTCTTCCTCTATAGCAAGCTCACCGTGGACAAGAGCAGGTGGCAGCAGGGGAACGTCTTCTCATGCTCCGTGATGCATGAGGCTCTGCACAACCACTACACGCAGAAGAGCCTCTCCCTGTCCCCTGGTAAACACGATGAGCTCTAG

(B) ZikaEnv:aghFc

ATGGCTCGCTCGTTTGGAGCTAACAGTACCGTTGTGTTGGCGATCATCTTCTTCGGTGAGTGATTTTCCGATCTTCTTCTCCGATTTAGATCTCCTCTACATTGTTGCTTAATCTCAGAACCTTTTTTCGTTGTTCCTGGATCTGAATGTGTTTGTTTGCAATTTCACGATCTTAAAAGGTTAGATCTCGATTGGTATTGACGATTGGAATCTTTACGATTTCAGGATGTTTATTTGCGTTGTCCTCTGCAGGATCCATACGATGTATTGGTGTTTCAAATAGGGATTTTGTTGAAGGCATGAGTGGAGGGACATGGGTGGACGTTGTATTGGAGCACGGCGGTTGCGTAACCGTGATGGCCCAAGATAAGCCAACTGTTGACATTGAACTGGTCACGACTACTGTGTCAAATATGGCTGAAGTGCGGAGCTATTGTTATGAAGCATCTATTAGTGATATGGCTAGTGATTCAAGATGTCCTACACAGGGAGAAGCATATCTGGATAAGCAGTCCGATACACAATATGTATGCAAACGTACTCTTGTTGATAGGGGTTGGGGTAACGGTTGCGGATTATTCGGCAAGGGTTCCTTGGTTACTTGTGCTAAATTCGCGTGTAGCAAAAAGATGACAGGTAAGTCGATACAGCCTGAGAATCTTGAGTACCGCATCATGTTGTCTGTTCATGGTAGTCAACATTCTGGGATGATCGTCAATGACACTGGGCATGAGACTGATGAAAACAGAGCAAAGGTTGAGATTACTCCCAATTCACCAAGGGCTGAAGCTACTCTCGGAGGATTTGGTTCTTTAGGATTGGATTGCGAGCCGAGAACTGGACTAGATTTTTCTGATCTTTACTACCTTACTATGAATAACAAACATTGGCTCGTTCACAAAGAATGGTTTCACGACATTCCACTTCCTTGGCATGCCGGAGCAGACACTGGTACTCCTCATTGGAATAACAAAGAGGCTCTGGTCGAATTTAAGGACGCTCACGCAAAAAGACAAACAGTTGTGGTTTTAGGTAGCCAGGAGGGGGCAGTTCATACGGCTTTGGCAGGTGCCTTGGAAGCTGAAATGGATGGTGCCAAGGGTCGATTGTCCAGTGGTCACTTGAAGTGTAGATTGAAGATGGATAAACTTCGTCTTAAGGGGGTGTCATATTCTCTGTGCACTGCTGCTTTTACTTTCACAAAAATACCGGCAGAAACGTTACATGGTACCGTGACAGTCGAGGTTCAATATGCTGGAACTGATGGGCCTTGTAAGGTACCAGCCCAGATGGCTGTCGATATGCAAACCTTAACACCAGTTGGAAGACTTATAACTGCTAATCCCGTTATTACAGAGTCTACTGAAAATTCTAAAATGATGCTAGAGCTTGATCCTCCATTTGGAGACTCTTACATTGTGATTGGAGTTGGTGAAAAAAAGATTACTCATCATTGGCATAGGTCTGGTAGTACAATCGGTAAAGCATTTGAAGCTACCGTAAGGGGCGCGAGAAGAATGGCTGTTCTTGGAGATACAGCTTGGGATTTTGGATCAGTAGGTGGAGCCCTCAACTCACTCGGAAAAGGTATTCACCAGATCTTCGGTGCAGCATTCAAGGCCCGGGGTGGGGGAGGCAGTGGCGGAGGTGGATCACCACCTTGCCCAGCTCCTGAATTGCTTGGAGGTCCTTCTGTTTTTCTTTTTCCACCTAAGCCAAAAGATACATTGATGATTTCTAGGACACCTGAGGTTACTTGCGTTGTTGTTGATGTTTCACATGAAGATCCAGAGGTTAAGTTTAATTGGTACGTTGATGGAGTTGAAGTTCATAATGCTAAGACTAAACCAAGGGAAGAGCAATACGCCTCTACATACAGAGTTGTTTCAGTTTTGACTGTTCTTCATCAAGATTGGCTTAACGGAAAGGAATACAAGTGTAAAGTTTCTAACAAGGCTTTGCCAGCTCCTATCGAAAAGACAATTTCAAAGGCTAAAGGTCAACCAAGGGAGCCTCAAGTTTACACTCTTCCACCATCAAGAGATGAATTGACAAAGAACCAAGTTTCATTGACTTGCCTTGTTAAGGGATTCTACCCTTCTGATATTGCTGTTGAATGGGAGTCAAACGGTCAACCAGAAAACAACTACAAGACTACACCACCTGTTCTTGATTCTGATGGATCTTTCTTTCTTTACTCTAAACTTACTGTTGATAAGTCAAGATGGCAACAGGGTAATGTTTTCTCTTGTTCAGTTATGCACGAGGCACTTCACAATCACTACACACAAAAATCTTTATCTTTATCACCTGGTAAAGATGAGCTCTAG
